# Characteristics of discordance between amyloid positron emission tomography and plasma amyloid-β 42/40 positivity

**DOI:** 10.1038/s41398-024-02766-6

**Published:** 2024-02-10

**Authors:** Jung-Min Pyun, Young Ho Park, Young Chul Youn, Min Ju Kang, Kyu Hwan Shim, Jae-Won Jang, Jihwan You, Kwangsik Nho, SangYun Kim, the Alzheimer’s Disease Neuroimaging Initiative, Michael W. Weiner, Paul Aisen, Ronald Petersen, Clifford R. Jack, William Jagust, John Q. Trojanowki, Arthur W. Toga, Laurel Beckett, Robert C. Green, Andrew J. Saykin, John Morris, Leslie M. Shaw, Greg Sorensen, Maria Carrillo, Lew Kuller, Marc Raichle, Steven Paul, Peter Davies, Howard Fillit, Franz Hefti, David Holtzman, M. Marcel Mesulam, William Potter, Peter Snyder, James Hendrix, Aparna Vasanthakumar, Tom Montine, Michael Rafii, Tiffany Chow, Rema Raman, Gustavo Jimenez, Michael Donohue, Devon Gessert, Kelly Harless, Jennifer Salazar, Yuliana Cabrera, Sarah Walter, Lindsey Hergesheimer, Danielle Harvey, Michael Donohue, Matthew Bernstein, Nick Fox, Paul Thompson, Norbert Schuff, Charles DeCArli, Bret Borowski, Jeff Gunter, Matt Senjem, Prashanthi Vemuri, David Jones, Kejal Kantarci, Chad Ward, Robert A. Koeppe, Norm Foster, Eric M. Reiman, Kewei Chen, Chet Mathis, Susan Landau, Nigel J. Cairns, Erin Franklin, Virginia Lee, Magdalena Korecka, Michal Figurski, Karen Crawford, Scott Neu, Tatiana M. Foroud, Steven Potkin, Li Shen, Kelley Faber, Sungeun Kim, Marilyn Albert, Richard Frank, John Hsiao, Zaven Khachaturian

**Affiliations:** 1 Department of Neurology, Soonchunhyang University Seoul Hospital, Soonchunhyang University College of Medicine, 59, Daesagwan-ro, Yongsan-gu, Seoul, 04401 Republic of Korea; 2 Department of Neurology, Seoul National University Bundang Hospital and Seoul National University College of Medicine, 82, Gumi-ro 173 Beon-gil, Bundang-gu, Seongnam-si, Gyeonggi-do 13620 Republic of Korea; 3 Department of Neurology, Chung-Ang University Hospital, 102, Heukseok-ro, Dongjak-gu, Seoul, 06973 Republic of Korea; 4 Department of Neurology, Veterans Health Service Medical Center, 53, Jinhwangdo-ro 61-gil, Gangdong-gu, Seoul, 05368 Republic of Korea; 5 Department of Bionano Technology, Gachon University, 1342, Seongnamdaero, Sujeong-gu, Seongnam-si, Gyeonggi-do 13120 Republic of Korea; 6 Department of Neurology, Kangwon National University Hospital, Kangwon National University College of Medicine, 156, Baengnyeong-ro, Chuncheon-si, Gangwon-do 24289 Republic of Korea; 7 Department of Radiology and Imaging Sciences, and the Indiana Alzheimer Disease Center, Indiana University School of Medicine, 355 W 16th, GH 4101, Indianapolis, IN 46202 USA; 8 Center for Computational Biology and Bioinformatics, Indiana University School of Medicine, 410 W 10th, Health Information and Translational Science Building, Suite 5000, Indianapolis, IN 46202 USA; 9 University of California San Francisco, San Francisco, CA USA; 10 University of Southern California, Los Angeles, CA USA; 11 Mayo Clinic, Rochester, MN USA; 12 University of California Berkeley, Berkeley, CA USA; 13 University of Pennsylvania, Philadelphia, PA USA; 14 University of California Davis, Davis, CA USA; 15 Brigham and Women’s Hospital/Harvard Medical School, Boston, MA USA; 16 Washington University St. Louis, St. Louis, MO USA; 17 Siemens, Charlotte, NC USA; 18 Alzheimer’s Association, Chicago, IL USA; 19 University of Pittsburgh, Pittsburgh, PA USA; 20 Cornell University, Ithaca, NY USA; 21 Albert Einstein College of Medicine of Yeshiva University, Bronx, NY USA; 22 AD Drug Discovery Foundation, New York, NY USA; 23 Acumen Pharmaceuticals, Charlottesville, VA USA; 24 Northwestern University, Evanston, IL USA; 25 National Institute of Mental Health, Bethesda, MD USA; 26 Brown University, Providence, RI USA; 27 AbbVie, JD Hoofddorp, Amsterdam, Netherlands; 28 University of Washington, Seattle, WA USA; 29 University of California San Diego, La Jolla, CA USA; 30 University of London, London, England UK; 31 UCLA School of Medicine, Los Angeles, CA USA; 32 University of Michigan, Ann Arbor, MI USA; 33 University of Utah, Salt Lake City, UT USA; 34 Banner Alzheimer’s Institute, Phoenix, AZ USA; 35 Indiana University, Bloomington, IN USA; 36 University of California Irvine, Irvine, CA USA; 37 Johns Hopkins University, Baltimore, MD USA; 38 Richard Frank Consulting, Gainesville, FL USA; 39 National Institute of Aging, Bethesda, MD USA; 40 Prevent Alzheimer’s Disease 2020, Bethesda, MD USA

**Keywords:** Molecular neuroscience, Diagnostic markers

## Abstract

Various plasma biomarkers for amyloid-β (Aβ) have shown high predictability of amyloid PET positivity. However, the characteristics of discordance between amyloid PET and plasma Aβ42/40 positivity are poorly understood. Thorough interpretation of discordant cases is vital as Aβ plasma biomarker is imminent to integrate into clinical guidelines. We aimed to determine the characteristics of discordant groups between amyloid PET and plasma Aβ42/40 positivity, and inter-assays variability depending on plasma assays. We compared tau burden measured by PET, brain volume assessed by MRI, cross-sectional cognitive function, longitudinal cognitive decline and polygenic risk score (PRS) between PET/plasma groups (PET−/plasma−, PET−/plasma+, PET+/plasma−, PET+/plasma+) using Alzheimer’s Disease Neuroimaging Initiative database. Additionally, we investigated inter-assays variability between immunoprecipitation followed by mass spectrometry method developed at Washington University (IP-MS-WashU) and Elecsys immunoassay from Roche (IA-Elc). PET+/plasma+ was significantly associated with higher tau burden assessed by PET in entorhinal, Braak III/IV, and Braak V/VI regions, and with decreased volume of hippocampal and precuneus regions compared to PET−/plasma-. PET+/plasma+ showed poor performances in global cognition, memory, executive and daily-life function, and rapid cognitive decline. PET+/plasma+ was related to high PRS. The PET−/plasma+ showed intermediate changes between PET−/plasma− and PET+/plasma+ in terms of tau burden, hippocampal and precuneus volume, cross-sectional and longitudinal cognition, and PRS. PET+/plasma− represented heterogeneous characteristics with most prominent variability depending on plasma assays. Moreover, IP-MS-WashU showed more linear association between amyloid PET standardized uptake value ratio and plasma Aβ42/40 than IA-Elc. IA-Elc showed more plasma Aβ42/40 positivity in the amyloid PET-negative stage than IP-MS-WashU. Characteristics of PET−/plasma+ support plasma biomarkers as early biomarker of amyloidopathy prior to amyloid PET. Various plasma biomarker assays might be applied distinctively to detect different target subjects or disease stages.

## Introduction

Alzheimer’s disease (AD) is characterized by pathological hallmarks with amyloidopathy and tauopathy leading to neuronal degeneration with cognitive impairment [1]. Amyloid pathology as the early change of AD, can be evaluated by amyloid plaque detection using amyloid positron emission tomography (PET) and cerebrospinal fluid (CSF) amyloid-β (Aβ) concentration measurement via a CSF study [1]. Several assays measuring plasma Aβ have been developed and showed promising performance to predict amyloid PET positivity [2, 3]. These plasma Aβ biomarkers have been validated in diverse cohorts and clinical use is expected, owing to cost advantages and practical value [4].

Plasma Aβ biomarker performance has often been assessed by the concordance rate with amyloid PET finding, which has been used as a gold standard biomarker for central amyloidopathy. Although plasma biomarkers have been optimized reaching high concordance rate, discordant cases between amyloid PET and plasma Aβ are needed to be understood regarding their characteristics. Discordance could originate from differences in detection target between PET and plasma biomarker and technical limitation of plasma assay. Discordant groups could also represent different disease stages, or presence of concomitant mixed pathology than concordant group. Inter-assay variability can also be considered. There are various assays to measure plasma Aβ with different methodologies [2]. Exploring whether discordant group in an assay is also discordant in another can help understand the group feature. As plasma biomarker Aβ is imminent to integration into clinical guidelines, proper interpretation of discordant case is important. To the best of our knowledge, no prior studies have explored the discordant cases.

This study aimed to investigate the characteristics of concordant and discordant groups between PET and plasma amyloid positivity (PET−/plasma−, PET−/plasma+, PET+/plasma−, PET+/plasma+). We compared tau burden measured by PET, brain volume assessed by magnetic resonance imaging (MRI), cross-sectional cognitive function, longitudinal cognitive decline, and polygenic risk score (PRS) between PET/plasma groups. We also compared the characteristics of groups classified by two different plasma assays. Aβ42/40 level measurement of identical samples by various plasma assays were used from Alzheimer’s Disease Neuroimaging Initiative (ADNI) database. We included two assays, immunoprecipitation followed by mass spectrometry (IP-MS) method developed at Washington University (IP-MS-WashU) and Elecsys immunoassay from Roche Diagnostics (IA-Elc), which showed the most promising amyloid PET predictability among IP-MS assays and immunoassays in previous head-to-head comparison study [3].

## Materials and methods

### Participants

We obtained data from the ADNI database (http://adni.loni.usc.edu). ADNI launched in 2003 as a public–private partnership, primarily aims to test whether neuroimaging, other biological markers, and clinical neuropsychological assessment can be combined to measure the progression of mild cognitive impairment (MCI) and early AD. Written informed consent was obtained at the time of enrollment and included permission for analysis and data sharing. The protocol and informed consent forms were approved by the institutional review boards at each the participating institution.

Here, we included participants based on the availability of amyloid PET and plasma Aβ42/40 profiles by two different assays, IP-MS-WashU and IA-Elc. Duplicate plasma measurements (*n* = 9) were excluded from both assays, and one sample with failed quality check was excluded from IP-MS-WashU. For analyses, we used 120 participants from IP-MS-WashU and 121 participants from IA-Elc.

### Neuroimaging

Amyloid imaging was acquired using [^18^F]florbetapir PET in four 5 min frames 50–70 min post injection of 10 mCi and spatially normalized to the statistical parametric mapping (SPM) template using SPM8 (Wellcome Trust Center for Neuroimaging, UCL, UK) in MATLAB R2013a (Mathworks, Natick, MA). Additional details of data processing are available online (http://adni.info.org). Standardized uptake value ratio (SUVR), summary value of florbetapir retention, was determined using the global cortical target region of interest (ROI) with the cerebellum reference regions [5]. Amyloid PET positivity was defined as SUVR ≥ 1.11 [6].

Tau imaging using Flortaucipir (FTP) PET was performed and co-registered to the MRI closest to the tau visit. The FTP SUVR was generated using inferior cerebellum gray matter as a reference region. We used composite Braak ROIs for the analysis, which approximate tau spreading in the anatomical Braak stages [7], Braak1 (entorhinal), Braak III/IV (III: parahippocampal, fusiform, lingual, and amygdala regions/ IV: middle temporal, caudal anterior cingulate, rostral anterior cingulate, posterior cingulate, isthmus cingulate, insula, inferior temporal, and temporal pole regions), and Braak V/VI (V: superior frontal, lateral orbitofrontal, medial orbitofrontal, frontal pole, caudal middle frontal, rostral middle frontal, pars opercularis, pars orbitalis, pars triangularis, lateral occipital, parietal supramarginal, parietal inferior, superior temporal, parietal superior, precuneus, bank of superior temporal sulcus, and transverse temporal regions/ VI: pericalcarine, postcentral, cuneus, precentral, and paracentral regions). Braak2 (hippocampus) was not included, since this region can be contaminated by off-target binding in the choroid plexus.

Structural brain volume was estimated from T1-weighted brain MRI scans using FreeSurfer (surfer.nmr.mgh.harvard.edu) [8]. ROIs of temporal and parietal regions including parahippocampus, hippocampus, precuneus, superior parietal, and inferior parietal lobes were adjusted for the estimated intracranial volume. More details regarding neuroimaging processing are available online (http://adni.info.org).

### Plasma Aβ42/40

ADNI blood sample were collected in two 10 mL EDTA tubes and centrifuged at room temperature within 1 h of collection. After centrifugation for 15 min at 1300 rpm, plasma samples were frozen and shipped to the Biomarker Core Laboratory. More details are available online (http://adni.info.org).

Plasma concentrations of Aβ42 and Aβ40 were analyzed using IP-MS-WashU and IA-Elc between December 2020 and March 2021. The optimal value of plasma Aβ42/40 to differentiate amyloid PET positivity was estimated among ADNI subjects based on the Youden index, using receiver operating characteristics (ROC) analysis (eFig. 1 in Supplement 1). The cutoffs were 0.1279 for IP-MS-WashU with area under the curve (AUC) value of 0.807 (95% confidence interval (CI) 0.726–0.888) and 0.1683 for IA-Elc with AUC value of 0.731 (95% CI 0.641–0.822). Plasma amyloid positivity was defined as Aβ42/40 ≤ 0.1279 for IP-MS-WashU and Aβ42/40 ≤ 0.1683 for IA-Elc.

### Cognition

Cognitive function was evaluated using Mini-Mental State Exam (MMSE) [9], Clinical Dementia Rating (CDR) [10], CDR Sum of Boxes (CDR SB), composite score of memory (ADNI MEM) [11] and executive function (ADNI EF) [12].

### PRS calculation

PRSs were calculated using the software PRSice v2.3.1.e [13]. The genome-wide association studies summary statistics from Jansen et al. were used as a base dataset [14] and the phase 3 genetic data from the 1000 Genomes Project [15] for non-Hispanic participants of European ancestry were used to calculate linkage disequilibrium structure. We used a *p*-value threshold of 1 × 10^−5^ to select AD-associated single nucleotide polymorphisms (SNPs). A total of 204 SNPs are used for PRS calculation. Furthermore, PRS was z-transformed based on the PRS distribution of amyloid PET-negative cognitively normal participants from the ADNI cohort.

### Statistical analyses

We classified participants into four groups according to PET and plasma amyloid positivity (PET−/plasma−, PET−/plasma+, PET+/plasma−, PET+/plasma+). Demographics between groups were compared using Kruskal–Wallis tests for continuous variables and chi-square tests for categorical variables. For multiple comparison correction a false discovery rate (FDR) of 0.05 using Benjamini-Hochberg procedure was used.

The distribution and concordance status of IP-MS-WashU and IA-Elc are displayed in scatterplots. To compare distribution between assays, plasma Aβ42/40 values were standardized to *z*-scores. Association between amyloid PET SUVR and plasma Aβ42/40 *z*-scores were depicted using smoothing spline curves. Concordance between IP-MS-WashU and IA-Elc were measured using Kendall’s tau-b correlation coefficient.

We performed association analyses of the PET/plasma groups with tau PET, brain MRI volumes, cross-sectional cognitive performances, and PRS using general linear model. Age and sex were adjusted in each models. Additionally, MRI field strength was adjusted in the analysis of brain MRI volume, and educational levels in the analysis of cognitive performance.

We performed association analysis between groups and longitudinal cognitive declines using linear mixed models adjusted for age, sex, and education levels. The variable of interest was the interaction of time and groups. The dependent variable was cognitive performance with the fixed effects being age, sex, and education levels and the random effect being subject.

We used the R software (version 4.1.3) for all analyses and statistical significance was set at *p* < 0.05. The R codes used in the study are available from the corresponding author upon reasonable request.

## Results

### Demographics of PET/plasma groups

Demographics of PET/plasma groups in both IP-MS-WashU and IA-Elc are shown in Table 1. In IP-MS-WashU, the majority was PET+/plasma+ (40.8%) followed by PET−/plasma− (37.5%). Among discordant groups (21.6%), PET−/plasma+ (13.3%) was larger than PET+/plasma− (8.3%). In IA-Elc, PET+/plasma+ was the majority (42.1%), followed by PET−/plasma− (28.9%). Discordant groups (28.9%) comprised larger PET−/plasma+ (22.3%) and smaller PET+/plasma− (6.6%).

**Table 1:** Demographics of study participants.

	IP-MS-WashU (*n* = 120)					IA-Elc (*n* = 121)				
	PET−/ plasma−	PET−/ plasma+	PET+/ plasma−	PET+/ plasma+	*p*-value	PET−/ plasma−	PET−/ plasma+	PET+/ plasma−	PET+/ plasma+	*p*-value
No. (%)	45 (38)	16 (13)	10 (8)	49 (41)		35 (29)	27 (22)	8 (7)	51 (42)	
Age, y	75 (72, 81)	77.5 (71, 79)	80 (77, 82)	79 (75, 84)	0.097	76 (72, 81)	77 (71, 80)	76 (74, 80)	80 (75, 83)	0.034
Male (%)	22 (49)	13 (81)	5 (50)	29 (59)	0.148	19 (54)	17 (63)	4 (50)	30 (59)	0.875
Education, y	18 (14, 18)	18 (15.5, 19)	18 (16, 18)	16 (12, 20)	0.557	18 (16, 19)	16 (14, 18)	15 (14, 18)	16 (13, 18)	0.207
*APOE* ε4 carrier (%)	11 (24)	8 (50)	3 (30)	26 (53)	0.026^a^	10 (29)	10 (37)	4 (50)	25 (49)	0.258
Time interval between PET and plasma, d	0 (−7.5, 2)	0 (−18, 2.5)	0 (−8, 1)	0 (−6, 4.5)	0.881	0 (−7.5, 2)	0 (−18, 2.5)	0 (−8, 1)	0 (−6, 4.5)	0.881
Plasma Aβ42/40	0.14 (0.13, 0.14)	0.12 (0.12, 0.12)	0.13 (0.13, 0.14)	0.12 (0.12, 0.12)	<0.001^b^	0.18 (0.17, 0.19)	0.15 (0.14, 0.16)	0.18 (0.17, 0.18)	0.15 (0.14, 0.16)	<0.001^d^
Amyloid PET SUVR	0.98 (0.91, 1.02)	1.02 (0.98, 1.06)	1.23 (1.16, 1.38)	1.33 (1.28, 1.47)	<0.001^c^	0.97 (0.93, 1.05)	1.01 (0.96, 1.02)	1.22 (1.15, 1.44)	1.33 (1.25, 1.46)	<0.001^e^

Abbreviations: *Aβ* amyloid-β, *IA-Elc* Elecsys immunoassay from Roche Diagnostics, *IP-MS-WashU* immunoprecipitation followed by mass spectrometry method developed at Washington, *IQR* interquartile range, *PET* positron emission tomography, *SUVR* standardized uptake value ratio.

Data are shown as median (IQR) unless otherwise specified.

Post-hoc analysis:

^a^*APOE* ε4 carrier: PET−/plasma− < PET+/plasma+: odds ratio = 3.44 [95% CI: 1.33–9.37], *p* = 0.036.

^b^Plasma Aβ42/40: PET−/plasma− < PET−/plasma+: Z = 5.746, *p* < 0.001, PET−/plasma+ < PET+/plasma−: Z = −3.89, *p* < 0.001, PET−/plasma− < PET+/plasma+: Z = 8.596, *p* < 0.001, PET+/plasma− < PET+/plasma+: Z = 4.72, *p* < 0.001.

^c^Amyloid PET SUVR: PET−/plasma− < PET+/plasma−: Z = −4.277, *p* < 0.001, PET−/plasma+ < PET+/plasma−: Z = −2.997, *p* = 0.004, PET−/plasma− < PET+/plasma+: Z = −9.020, *p* < 0.001, PET−/plasma+ < PET+/plasma+: Z = −5.470, *p* < 0.001.

^d^Plasma Aβ42/40: PET−/plasma− < PET−/plasma+: Z = 6.670, *p* < 0.001, PET−/plasma+ < PET+/plasma−: Z = −3.961, *p* < 0.001, PET−/plasma− < PET+/plasma+: Z = 8.045, *p* < 0.001, PET+/plasma− < PET+/plasma+: Z = 4.344, *p* < 0.001.

^e^Amyloid PET SUVR: PET−/plasma− < PET+/plasma−: Z = −3.988, *p* < 0.001, PET−/plasma+ < PET+/plasma−: Z = −3.708, *p* < 0.001, PET−/plasma− < PET+/plasma+: Z = −8.135, *p* < 0.001, PET−/plasma+ < PET+/plasma+: Z = −7.208, *p* < 0.001.

Both assays showed similar sex ratio, education levels, and same time interval between PET and plasma assessment. In IP-MS-WashU, PET+/plasma+ showed greater proportion of *APOE* ε4 carrier than PET-/plasma-. In IA-Elc, age was significantly different across the PET/plasma groups.

### Disagreement between IP-MS-WashU and IA-Elc

**Fig. 1:**
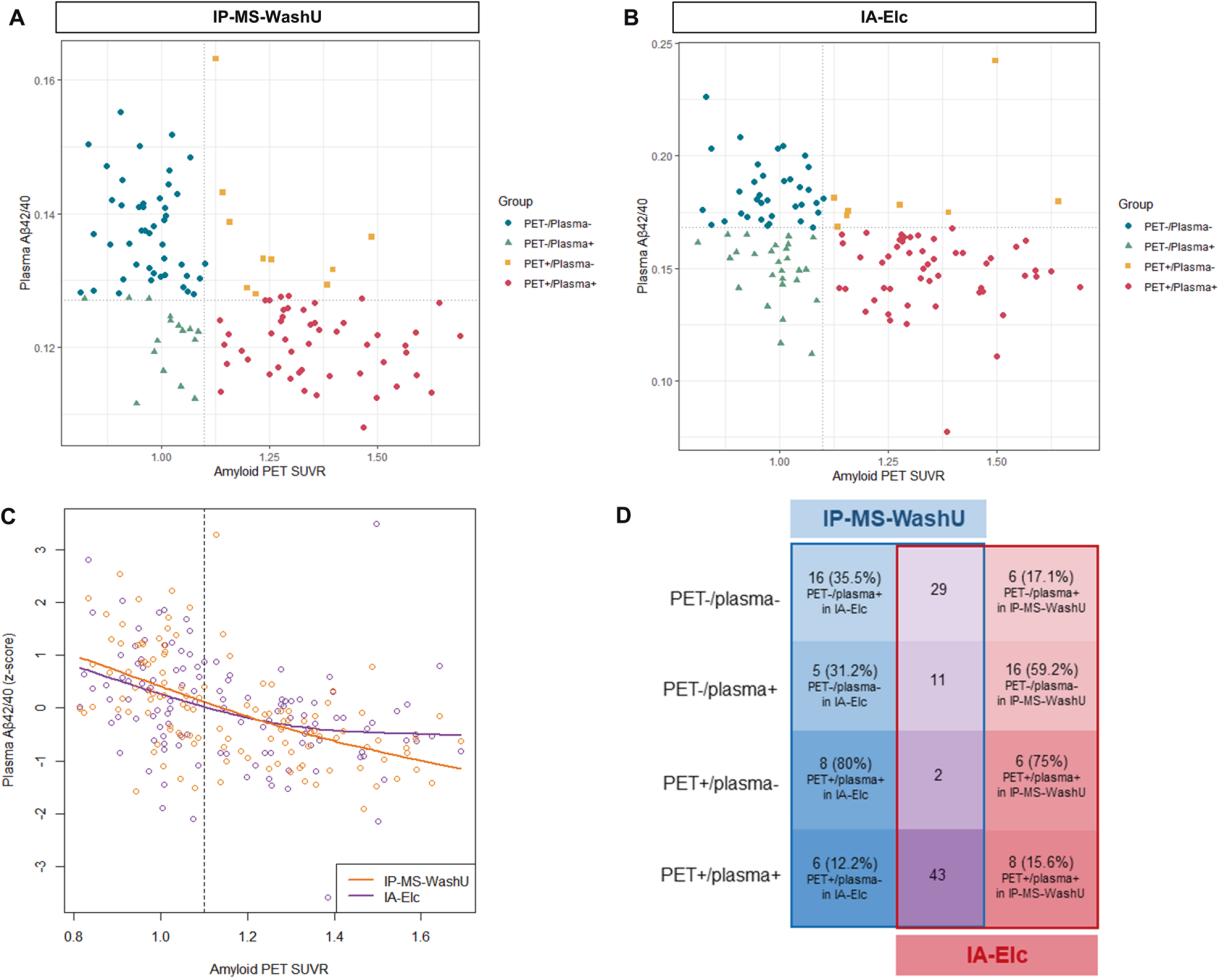
Scatterplots of distribution and concordance status between amyloid PET and plasma Aβ42/40. Distribution and concordance status of IP-MS-WashU (**A**) and IA-Elc (**B**). Plasma Aβ42/40 *z*-scores and amyloid PET SUVR values of IP-MS-WashU (orange) and IA-Elc (purple) are fitted using spline model (**C**). Disagreement number (%) between IP-MS-WashU (blue) and IA-Elc (red) were listed according to PET/plasma groups (**D**). Agreement cases between IP-MS-WashU and IA-Elc are listed in the middle (purple). Abbreviation: Aβ amyloid-β, IA-Elc Elecsys immunoassay from Roche Diagnostics, IP-MS-WashU immunoprecipitation followed by mass spectrometry method developed at Washington, PET positron emission tomography.

Distribution and concordance status of PET/plasma groups in IP-MS-WashU and IA-Elc are depicted in scatterplots (Fig. 1A, B). Plasma Aβ42/40 *z*-scores between IP-MS-WashU and IA-Elc were compared using smoothing spline curves, according to amyloid PET SUVR (Fig. 1C). Compared to IA-Elc, IP-MS-WashU showed more linear association between amyloid PET SUVR and plasma Aβ42/40 *z*-scores. In the amyloid-negative PET status, IA-Elc revealed plasma amyloid abnormality prior to IM-PS-WashU. The number of concordant and discordant cases between IP-MS-WashU and IA-Elc are presented in Fig. 1D. Kendall’s tau-b correlation coefficient between IP-MS-WashU and IA-Elc was 0.796 (*p* < 0.001). The highest agreement rate between IP-MS-WashU and IA-Elc was PET+/plasma+ and the highest disagreement rate between assays was observed in PET+/plasma− (IP-MS-WashU: 80%, IA-Elc: 75%).

### Association between PET/plasma groups and tau burden

**Fig. 2:**
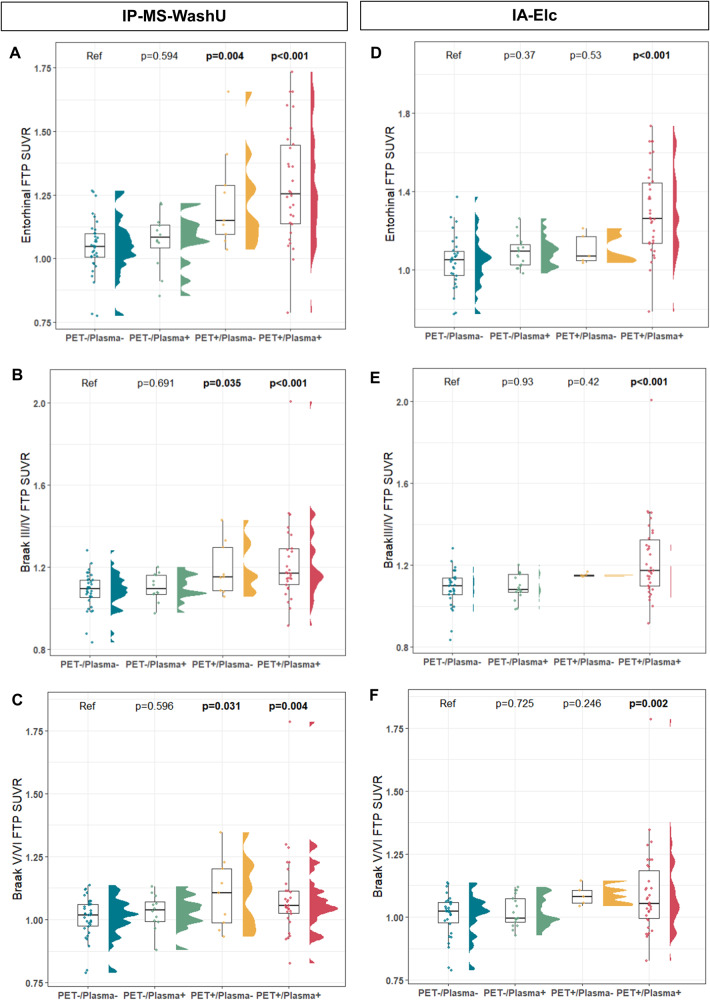
Tau burden according to PET/plasma groups. Association between tau burden assessed by18F-flortaucipir PET and PET/plasma groups were analyzed using general linear model adjusted for age and sex. Tau deposition in entorhinal, Braak III/IV, and Braak V/VI regions using IP-MS-WashU (**A**–**C**) and IA-Elc (**D**–**F**). Abbreviation: Aβ amyloid-β, FTP 18F-flortaucipir, IA-Elc Elecsys immunoassay from Roche Diagnostics, IP-MS-WashU immunoprecipitation followed by mass spectrometry method developed at Washington, PET positron emission tomography, SUVR standardized uptake value ratio.

PET+/plasma+ was significantly associated with higher tau PET uptake in entorhinal (IP-MS-WashU: estimate = 0.247, *p* < 0.001, IA-Elc: estimate = 0.266, *p* < 0.001), Braak III/IV (IP-MS-WashU: estimate = 0.145, *p* < 0.001, IA-Elc: estimate = 0.148, *p* < 0.001), and Braak V/VI regions (IP-MS-WashU: estimate = 0.091, *p* = 0.005, IA-Elc: estimate = 0.098, *p* = 0.002) in both assays compared to PET−/plasma− as a reference group (Fig. 2). In IP-MS-WashU, PET+/plasma− presented a significantly higher tau burden in entorhinal (estimate = 0.19, *p* = 0.004), Braak III/IV (estimate = 0.115, *p* = 0.035), and Braak V/VI regions (estimate = 0.102, *p* = 0.031) compared to PET−/plasma−. Overall, median SUVR values showed ascending tendency in the order of PET−/plasma−, PET−/plasma+, and PET+/plasma+. In contrast, PET+/plasma− revealed higher median SUVR value in Braak V/VI regions than even PET+/plasma+ (eTable 1 in Supplement 1).

### Association between PET/plasma groups and brain volume

**Fig. 3:**
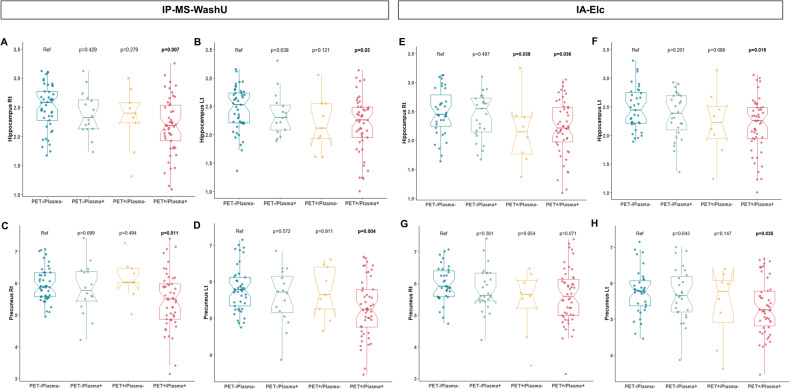
Hippocampal and precuneus volume according to PET/plasma groups. Association between brain volume assessed by MRI and PET/plasma groups were analyzed using general linear model adjusted for age, sex, estimated intracranial volume and MRI field strength. Bilateral hippocampal (**A**, **B**) and precuneus (**C**, **D**) volume according to PET/plasma groups using IP-MS-WashU and bilateral hippocampal (**E**, **F**) and precuneus (**C**, **D**) volume according to PET/plasma groups using IA-Elc (**G**, **H**). Abbreviations: IA-Elc Elecsys immunoassay from Roche Diagnostics, IP-MS-WashU immunoprecipitation followed by mass spectrometry method developed at Washington, Lt left, MRI magnetic resonance image, PET positron emission tomography, Rt right.

The association analyses of PET/plasma groups with brain volume included a comparison of parahippocampal, hippocampal, precuneus, supraparietal, and infraparietal regions as measured by MRI (eTable 2 in Supplement 1). Among the regions, hippocampus and precuneus showed consistent differences between groups in both assays. PET+/plasma+ showed a significantly low volume in the right (IP-MS-WashU: estimate = −0.236, *p* = 0.007, IA-Elc: estimate = −0.196, *p* = 0.036) and left (IP-MS-WashU: estimate = −0.188, *p* = 0.03, IA-Elc: estimate = −0.223, *p* = 0.015) hippocampus compared to PET−/plasma− in both assays (Fig. 3). In IA-Elc, PET+/plasma− also presented significant atrophy in right hippocampus (estimate = −0.341, *p* = 0.038) compared to PET−/plasma−. Precuneus region, which is part of AD-related parietal lobe atrophy, was observed in PET+/plasma+ compared PET−/plasma− in IP-MS-WashU (right precuneus: estimate = −0.388, *p* = 0.011; left precuneus: estimate = −0.418, *p* = 0.004). In IA-Elc, PET+/plasma+ showed significant lower volume in left precuneus (estimate = −0.335, *p* = 0.035) compared to PET−/plasma−.

### Association between PET/plasma groups and cross-sectional cognitive performance

**Fig. 4:**
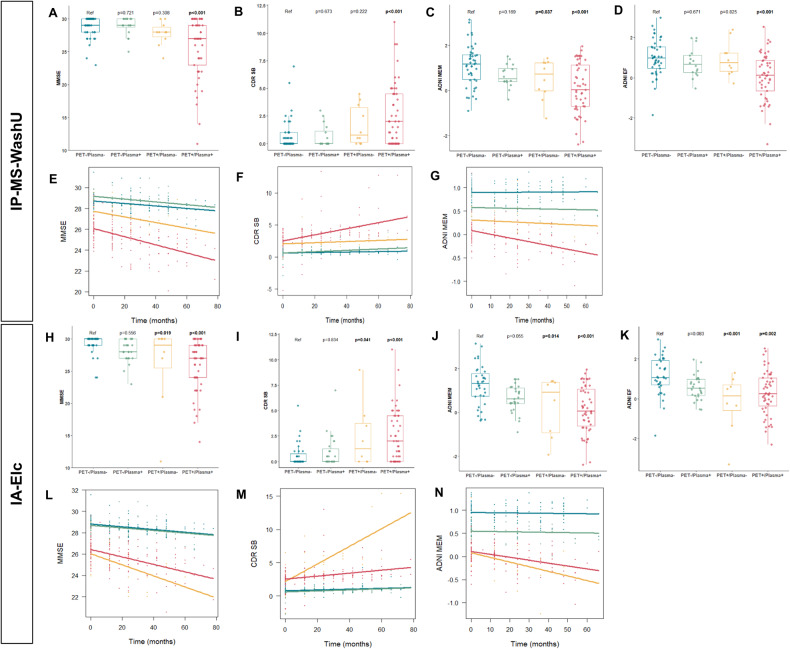
Cross-sectional cognitive performance and longitudinal cognitive decline according to PET/plasma groups. Association between cross-sectional cognitive performance and PET/plasma groups were analyzed using general linear model adjusted for age, sex, and education levels. MMSE, CDR SB, ADNI MEM, and ADNDI EF scores according to PET/plasma groups using IP-MS-WashU (**A**–**D**) and IA-Elc (**H**–**K**). Association between longitudinal cognitive changes and PET/plasma groups were analyzed linear mixed model adjusted for age, sex, and education levels. MMSE, CDR SB, and ADNI MEM changes according to PET/plasma groups using IP-MS-WashU (**E**–**G**) and IA-Elc (**L**–**N**). Abbreviations: ADNI EF Alzheimer’s Disease Neuroimaging Initiative composite score of executive function, ADNI MEM Alzheimer’s Disease Neuroimaging Initiative composite score of memory, CDR SB clinical dementia rating sum of boxes, IA-Elc Elecsys immunoassay from Roche Diagnostics, IP-MS-WashU immunoprecipitation followed by mass spectrometry method developed at Washington, MMSE mini mental state examination, PET positron emission tomography.

Cross-sectional cognitive performances assessed by MMSE, CDR SB, ADNI MEM, and ADNI EF between PET/plasma groups were compared (eTable 3 in Supplement 1). Association analyses showed that PET+/plasma+ showed lower MMSE (estimate = −2.661, *p* < 0.001), higher CDR (estimate = 0.274, *p* < 0.001), higher CDR SB (estimate = 1.713, *p* < 0.001), lower ADNI MEM (estimate = −0.834, *p* < 0.001) and lower ADNI EF scores (estimate = −0.689, *p* < 0.001) compared to PET−/plasma− in IP-MS-WashU (Fig. 4). Moreover, PET+/plasma− was associated with lower ADNI MEM score (estimate = −0.647, *p* = 0.037) than PET−/plasma−. In IA-Elc, PET+/plasma+ was related to significantly poor performances in MMSE (estimate = −2.56, *p* < 0.001), CDR (estimate = 0.287, *p* < 0.001), CDR SB (estimate = 1.676, *p* < 0.001), ADNI MEM (estimate = −0.891, *p* < 0.001), and ADNI EF (estimate = −0.61, *p* = 0.002) compared to the reference group. PET+/plasma− was also associated with significantly poor performances in MMSE (estimate = −2.928, *p* = 0.019), CDR SB (estimate = 1.701, *p* = 0.041), ADNI MEM (estimate = −0.853, *p* = 0.014), and ADNI EF/ (estimate = −1.25, *p* < 0.001) compared to the reference group.

### Association between PET/plasma groups and longitudinal cognitive decline

Associations of PET/plasma groups with longitudinal cognitive decline were assessed using linear mixed model. In IP-MS-WashU, significant interactions between groups and follow-up time were observed in MMSE (estimate = −0.009, *p* = 0.001), CDR (estimate = 0.001, *p* = 0.002), CDR SB (estimate = 0.014, *p* < 0.001) and ADNI MEM (estimate = −0.001, *p* = 0.001) (Fig. 4). In IA-Elc, significant interactions between groups and follow-up time were observed in MMSE (estimate = −0.008, *p* = 0.004), CDR SB (estimate = 0.009, *p* = 0.009), and ADNI MEM (estimate = −0.002, *p* = 0.007). In IA-Elc, PET+/plasma− showed the most rapid cognitive decline compared to other groups, which was not observed in IP-MS-WashU.

### Association between PET/plasma groups and PRS

Genetic data generating PRS were available only for 64 participants. Association analyses revealed that PET+/plasma+ was related with higher PRS both in IP-MS-WashU (estimate = 0.856, *p* = 0.008) and IA-Elc (estimate = 0.709, *p* = 0.043) (eFig. 2 in Supplement 1). The median values of PRS showed an ascending pattern across PET−/plasma−, PET−/plasma+, and PET+/plasma. However, PET+/plasma− represented the lowest median value in IP-MS-WashU and the highest in IA-Elc among other PET/plasma groups (eTable 4 in Supplement 1).

## Discussion

In this study, we investigated tau burden, brain volume, cross-sectional and longitudinal cognitive function, and PRS between groups defined by amyloid PET and plasma Aβ42/40 positivity.

In neuroimaging analysis, PET+/plasma+ were associated with higher tau PET SUVR values in entorhinal, Braak III/IV, and Braak V/VI regions and decreased volume in hippocampus and precuneus compared to PET−/plasma−. While tau burden and hippocampal and precuneus atrophy showed gradual changes across PET−/plasma−, PET−/plasma+, and PET+/plasma+, PET+/plasma− did not follow that order. In cognition analysis, PET+/plasma+ was associated with poor cognitive performance, and gradual decline pattern was shown across PET−/plasma−, PET−/plasma+, PET+/plasma−, and PET+/plasma+. Longitudinal cognitive performance declined in the order of PET−/plasma−, PET−/plasma+, and PET+/plasma+, whereas PET+/plasma− presented inconsistent pattern depending on plasma assays. PRS also showed variability in PET+/plasma−, whereas the ascending tendency was observed in order of PET−/plasma−, PET−/plasma+, and PET+/plasma+.

Overall, PET−/plasma+ showed intermediate changes between PET−/plasma− and PET+/plasma+ in terms of tau burden, hippocampal and precuneus volume, cognitive function, cognitive decline, and genetic risk score. In contrary, PET+/plasma− did not follow a specific order and instead, displayed heterogeneous characteristics with high tau burden in Braak V/VI regions, hippocampal atrophy, rapid cognitive decline, and diverse PRS depending on plasma biomarker assays. Although PET+/plasma− had the smallest proportion among PET/plasma groups within the assay (IP-MS-WashU: 8.3%, IA-Elc: 6.6%), highest disagreement rate was observed between assays (IP-MS-WashU: 80%, IA-Elc: 75%). This indicates that PET+/plasma− comprise heterogeneous characteristics. This might come from concomitant mixed pathology, different disease phenotypes, altered peripheral amyloid clearance capacity affected by other medical condition or medication, and difference in detection technology of plasma biomarkers. Careful investigation of these individuals might provide more helpful insight on the discordance.

Several previous studies reported the discordance between CSF and PET amyloidopathy positivity, showing abnormal changes in CSF prior to PET [16–20]. The discordant groups are considered to be intermediate between PET−/CSF− and PET+/CSF+ groups [19, 20]. PET−/CSF+ and PET+/CSF− are assumed to be two different Aβ processing pathways of the early amyloidopathy stages [20, 21]. Plasma biomarker as a fluid biomarker like CSF also undergoes abnormal changes prior to PET [22]. In our study, the PET−/plasma+ group tended to be in the intermediate state between PET−/plasma− and PET+/plasma+ in tau burden, brain volume, clinical course, and PRS, which supports the hypothesis of plasma biomarker being an early biomarker of amyloidopathy prior to PET. On the contrary, PET+/plasma− group was hardly the early stage of amyloidopathy. The difference between PET+/plasma− and PET+/CSF− might be because CSF and plasma Aβ are two distinct fluid biomarkers from central and peripheral systems. Plasma, which is easily affected by whole body system condition, might have different Aβ metabolism. Approximate use of two fluid biomarkers should be cautioned and comparison between CSF and plasma biomarkers should be explored in further studies.

Our results demonstrated inter-assays variabilities between IP-MS-WashU and IA-Elc. Regarding the distribution of PET/plasma groups, IA-Elc showed a higher proportion of PET−/plasma+, which was classified as PET−/plasma− measured by IP-MS-WashU (11/16, 59.2%). Moreover, 35.5% of PET−/plasma− (16/45) in IP-MS-WashU was defined as PET−/plasma+ in IA-Elc. This suggests that IA-Elc can detect more plasma amyloid abnormality in the early phase of disease with amyloid PET negativity. In contrast, IP-MS-WashU displayed a more linear association between plasma Aβ42/40 and amyloid PET SUVR showing higher amyloid PET positivity predictability. Furthermore, inter-assay differences in tau, brain volume, cognition, and PRS were observed in PET+/plasma−. These discrepancies indicate that two assays might detect individuals with distinct features and can be applied in different disease stages or target subjects according to characteristics of assays. To accomplish this, exploring relation of plasma biomarkers with different AD phenotypes, mixed pathologies, concomitant medical conditions, or medications would be helpful in the future.

### Limitations

This study had some limitations. First, the sample size was small and further studies with larger sample size are needed. However, this dataset, namely amyloid PET and plasma measurement of same sample at the same period using various assays, was optimal for the direct comparison between assays. Secondly, we defined thresholds for plasma amyloid positivity in the ADNI cohort based on cut-off values predictive of amyloid PET positivity. Due to the lack of standardized cut-off values for plasma assays, we employed the optimal values that best predict central amyloidopathy. However, it’s important to note that PET/plasma mismatch cases may vary depending on the chosen cut-off values, an issue that bears significance for both research and clinical applications. To address this, future study with diverse and large cohorts is essential to develop standardized cut-off values for plasma assays. Third, brain amyloidopathy was evaluated with [^18^F]florbetapir PET. Central amyloidopathy assessed by amyloid PET with other ligands or CSF measurement might also be meaningful. Fourth, subjects in the study showed relatively good cognitive performance (median CDR 0–0.5). Subjects with diverse cognitive stages might be needed. Fifth, pathological evidence would help better understand the characteristics of groups.

In this study, we found that gradual changes across PET−/plasma−, PET−/plasma+, and PET+/plasma+ regarding tau burden, brain volume, cognitive function, cognitive decline, and PRS could support plasma biomarkers as early biomarker of amyloidopathy prior to amyloid PET. PET+/plasma− showed heterogeneous characteristics. IP-MS-WashU and IA-Elc assays have different features and might be applied distinctively, detecting different target subjects or disease stages.

### Supplementary information

Supplementary table 1. Tau PET SUVR comparison of PET/plasma groups in IP-MS-WashU and IA-Elc

Supplementary table 2. Brain volume comparison of PET/plasma groups in IP-MS-WashU and IA-Elc

Supplementary table 3. Cross-sectional cognitive performance comparison of PET/plasma groups

Supplementary table 4. PRS comparison of PET/plasma groups

Supplementary figure 1. Receiver operating characteristics curve analyses for amyloid PET positivity predictability of IP-MS-WashU and IA-Elc

Supplementary figure 2. PRS according to PET/plasma groups

## Data Availability

All demographic, imaging, and genetic data in this study were publicly available and downloaded from the ADNI database (http://adni.info.org).
