# Supplementary material for: Characteristics of discordance between amyloid positron emission tomography and plasma amyloid-β 42/40 positivity
Source: Transl Psychiatry. 2024 Feb 10;14:88. doi: 10.1038/s41398-024-02766-6 (PMC10858862; doi:10.1038/s41398-024-02766-6)
Supplement: Supplementary file 1 — Supplementary table 1. Tau PET SUVR comparison of PET/plasma groups in IP-MS-WashU and IA-Elc [file 41398_2024_2766_MOESM1_ESM.docx]

Supplementary table 1. Tau PET SUVR comparison of PET/plasma groups in IP-MS-WashU and IA-Elc

|  | IP-MS-WashU | | | | | IA-Elc | | | | |
| --- | --- | --- | --- | --- | --- | --- | --- | --- | --- | --- |
| FTP PET (SUVR)^a^ | PET-/  plasma- | PET-/  plasma+ | PET+/  plasma- | PET+/  plasma+ | *p*-value | PET-/  plasma- | PET-/  plasma+ | PET+/  plasma- | PET+/  plasma+ | *p*-value |
| Entorhinal | 1.05  (1.01, 1.19) | 1.08  (1.02, 1.14) | 1.15  (1.09, 1.29) | 1.25  (1.14, 1.45) | <0.001^b^ | 1.05  (0.97, 1.10) | 1.09  (1.02, 1.13) | 1.07  (1.05, 1.17) | 1.26  (1.14, 1.45) | < 0.001^d^ |
| Braak III/IV | 1.09  (1.05, 1.14) | 1.09  (1.07, 1.16) | 1.15  (1.09, 1.30) | 1.17  (1.11, 1.29) | 0.002^c^ | 1.10  (1.06, 1.14) | 1.08  (1.07, 1.15) | 1.15  (1.15, 1.15) | 1.17  (1.10, 1.33) | 0.002^e^ |
| Braak V/VI | 1.02  (0.97, 1.06) | 1.04  (0.99, 1.08) | 1.11  (0.99, 1.20) | 1.06  (1.02, 1.11) | 0.065 | 1.02  (0.97, 1.06) | 0.99  (0.98, 1.07) | 1.08  (1.06, 1.11) | 1.05  (0.99, 1.20) | 0.042 |

Data are shown as median (IQR).

^a^Tau PET of 35 participants were not available.

Post-hoc analysis:

^b^Entorhinal FTP SUVR: PET-/plasma- < PET+/plasma-: Z = -2.736, *p* = 0.012; PET-/plasma- < PET+/plasma+: Z = -4.885, *p* < 0.001; PET-/plasma+ < PET+/plasma+: Z = -2.856, *p* = 0.012

^c^Braak III/IV FTP SUVR: PET-/plasma- < PET+/plasma+: Z = -3.568, *p* = 0.002

^d^Entorhinal FTP SUVR: PET-/plasma- < PET+/plasma+: Z = -5.222, *p* < 0.001; PET-/plasma+ < PET+/plasma+: Z = -3.283, *p* = 0.003

^e^Braak III/IV FTP SUVR: PET-/plasma- < PET+/plasma+: Z = -3.366, *p* = 0.004; PET-/plasma+ < PET+/plasma+: Z = -2.914, *p* = 0.010

Abbreviations: FTP, 18F-flortaucipir; IA-Elc, Elecsys immunoassay from Roche Diagnostics; IP-MS-WashU, immunoprecipitation followed by mass spectrometry method developed at Washington; IQR, interquartile range; PET, positron emission tomography; SUVR, standardized uptake value ratio.
