# Supplementary material for: Characteristics of discordance between amyloid positron emission tomography and plasma amyloid-β 42/40 positivity
Source: Transl Psychiatry. 2024 Feb 10;14:88. doi: 10.1038/s41398-024-02766-6 (PMC10858862; doi:10.1038/s41398-024-02766-6)
Supplement: Supplementary file 2 — Supplementary table 2. Brain volume comparison of PET/plasma groups in IP-MS-WashU and IA-Elc [file 41398_2024_2766_MOESM2_ESM.docx]

Supplementary table 2. Brain volume comparison of PET/plasma groups in IP-MS-WashU and IA-Elc

|  | IP-MS-WashU | | | | | IA-Elc | | | | |
| --- | --- | --- | --- | --- | --- | --- | --- | --- | --- | --- |
| MRI | PET-/  plasma- | PET-/  plasma+ | PET+/  plasma- | PET+/  plasma+ | *p*-value | PET-/  plasma- | PET-/  plasma+ | PET+/  plasma- | PET+/  plasma+ | *p*-value |
| Parahippocampus rt | 1.30  (1.18, 1.47) | 1.28  (1.13, 1.43) | 1.32  (1.04, 1.47) | 1.22  (1.01, 1.32) | 0.08 | 1.29  (1.14, 1.41) | 1.31  (1.18, 1.46) | 1.14  (0.97, 1.23) | 1.24  (1.01, 1.35) | 0.046 |
| Parahippocampus lt | 1.37  (1.18, 1.58) | 1.34  (1.17, 1.57) | 1.28  (1.00, 1.49) | 1.23  (1.07, 1.39) | 0.029^a^ | 1.36  (1.15, 1.56) | 1.42  (1.20, 1.58) | 1.19  (1.05, 1.38) | 1.25  (1.07, 1.4) | 0.034 |
| Hippocampus rt | 2.58  (2.28, 2.77) | 2.32  (2.13, 2.64) | 2.40  (2.24, 2.58) | 2.18  (1.93, 2.54) | 0.012^b^ | 2.45  (2.25, 2.79) | 2.55  (2.15, 2.73) | 2.14  (1.74, 2.41) | 2.21  (1.9, 2.58) | 0.035 |
| Hippocampus lt | 2.53  (2.21, 2.74) | 2.30  (2.07, 2.53) | 2.11  (1.94, 2.55) | 2.26  (1.95, 2.48) | 0.041^c^ | 2.44  (2.21, 2.75) | 2.38  (2.09, 2.69) | 2.22  (1.88, 2.56) | 2.26  (1.94, 2.49) | 0.065 |
| Precuneus rt | 5.88  (5.57, 6.33) | 5.77  (5.39, 6.38) | 6.03  (6.02, 6.47) | 5.50  (4.87, 5.99) | 0.008^d^ | 5.91  (5.60, 6.40) | 5.63  (5.46, 6.33) | 5.66  (4.92, 6.17) | 5.60  (5.00, 6.15) | 0.13 |
| Precuneus lt | 5.78  (5.33, 6.12) | 5.71  (5.12, 6.19) | 5.64  (5.25, 6.40) | 5.25  (4.75, 5.79) | 0.013^e^ | 5.78  (5.37, 6.07) | 5.65  (5.20, 6.18) | 5.77  (4.65, 6.14) | 5.25  (4.77, 5.79) | 0.053 |
| Supraparietal rt | 7.74  (7.32, 8.39) | 7.47  (6.88, 7.81) | 7.87  (7.37, 7.94) | 7.17  (6.70, 7.85) | 0.044^f^ | 7.74  (7.30, 8.41) | 7.49  (6.80, 7.93) | 7.37  (6.99, 7.72) | 7.36  (6.72, 7.94) | 0.151 |
| Supraparietal lt | 7.67  (7.25, 8.43) | 7.77  (6.88, 7.96) | 7.25  (7.04, 8.66) | 7.34  (6.75, 8.22) | 0.351 | 7.68  (7.23, 8.43) | 7.63  (7.04, 8.02) | 7.18  (6.95, 8.22) | 7.41  (6.75, 8.23) | 0.537 |
| Infraparietal rt | 8.83  (8.22, 9.54) | 8.14  (7.35, 9.59) | 8.57  (7.99, 9.44) | 8.61  (7.69, 9.45) | 0.387 | 8.93  (8.28, 9.54) | 8.30  (7.71, 9.28) | 8.71  (7.79, 9.31) | 8.52  (7.74, 9.45) | 0.344 |
| Infraparietal lt | 7.30  (6.72, 7.80) | 6.59  (6.04, 7.13) | 7.14  (6.92, 7.87) | 6.86  (6.27, 7.30) | 0.004^g^ | 7.20  (6.65, 7.57) | 7.05  (6.23, 7.68) | 6.78  (5.98, 7.20) | 6.93  (6.31, 7.38) | 0.278 |

Data are shown as median (IQR).

Post-hoc analysis:

^a^Parahippocampus lt: PET-/plasma- < PET+/plasma+: Z = 2.909, *p* = 0.021

^b^Hippocampus rt: PET-/plasma- < PET+/plasma+: Z = 3.312, *p* = 0.005

^c^Hippocampus lt: PET-/plasma- < PET+/plasma+: Z = 2.699, *p* = 0.041

^d^Precuneus rt: PET-/plasma- < PET+/plasma+: Z = 2.877, *p* = 0.024; PET+/plasma- < PET+/plasma+: Z = 2.562, *p* = 0.031

^e^Precuneus lt: PET-/plasma- < PET+/plasma+: Z = 3.191, *p* = 0.008

^f^Supraparietal rt: PET-/plasma- < PET+/plasma+: Z = 2.668, *p* = 0.045

^g^Infraparietal lt: PET-/plasma- < PET+/plasma+: Z = 3.045, *p* = 0.013; PET-/plasma- < PET-/plasma+: Z = 2.631, *p* = 0.025

Abbreviations: IA-Elc, Elecsys immunoassay from Roche Diagnostics; IP-MS-WashU, immunoprecipitation followed by mass spectrometry method developed at Washington; IQR, interquartile range; lt, left; MRI, Magnetic resonance image; PET, positron emission tomography; rt, right.
