# Supplementary material for: Characteristics of discordance between amyloid positron emission tomography and plasma amyloid-β 42/40 positivity
Source: Transl Psychiatry. 2024 Feb 10;14:88. doi: 10.1038/s41398-024-02766-6 (PMC10858862; doi:10.1038/s41398-024-02766-6)
Supplement: Supplementary file 3 — Supplementary table 3. Cross-sectional cognitive performance comparison of PET/plasma groups [file 41398_2024_2766_MOESM3_ESM.docx]

Supplementary table 3. Cross-sectional cognitive performance comparison of PET/plasma groups

|  | IP-MS-WashU | | | | | IA-Elc | | | | |
| --- | --- | --- | --- | --- | --- | --- | --- | --- | --- | --- |
| Cognition | PET-/  plasma- | PET-/  plasma+ | PET+/  plasma- | PET+/  plasma+ | *p*-value | PET-/  plasma- | PET-/  plasma+ | PET+/  plasma- | PET+/  plasma+ | *p*-value |
| MMSE | 29  (28, 30) | 29  (28.5, 30) | 28  (27, 29) | 27  (23, 29) | 0.001^a^ | 30  (29, 30) | 28  (27, 30) | 29  (24, 30) | 27  (24, 29) | <0.001^f^ |
| CDR | 0  (0, 0.5) | 0  (0, 0.5) | 0.5  (0, 0.5) | 0.5  (0, 1) | 0.001^b^ | 0  (0, 0.5) | 0  (0, 0.5) | 0.5  (0, 0.75) | 0.5  (0, 1) | 0.001^g^ |
| CDR SB | 0  (0, 1) | 0  (0, 1.25) | 0.75  (0, 3.5) | 2  (0, 4.5) | 0.001^c^ | 0  (0, 0.75) | 0  (0, 1.25) | 1.25  (0.00, 4.00) | 2  (0, 4.50) | 0.001^h^ |
| ADNI MEM | 1.16  (0.48, 1.58) | 0.52  (0.38, 1.04) | 0.72  (-0.05, 1.23) | 0.02  (-0.7, 1.12) | <0.001^d^ | 1.32  (0.74, 1.78) | 0.61  (0.40, 1.11) | 0.91  (-1.00, 1.38) | 0.05  (-0.63, 1.05) | <0.001^i^ |
| ADNI EF | 0.98  (0.46, 1.53) | 0.66  (0.23, 1.15) | 0.74  (0.27, 1.22) | 0.12  (-0.66, 0.87) | 0.001^e^ | 1.06  (0.70, 1.93) | 0.52  (0.17, 0.98) | 0.13  (-0.85, 0.79) | 0.24  (-0.37, 1.04) | <0.001^j^ |

Data are shown as median (IQR).

Post-hoc analysis:

^a^MMSE: PET-/plasma- < PET+/plasma+: Z = 3.789, *p* = 0.001; PET-/plasma+ < PET+/plasma+: Z = 2.668, *p* = 0.022

^b^CDR: PET-/plasma- < PET+/plasma+: Z = -3.609, *p* = 0.001; PET-/plasma+ < PET+/plasma+: Z = -2.751, *p* = 0.017

^c^CDR SB: PET-/plasma- < PET+/plasma+: Z = -3.793, *p* = 0.001; PET-/plasma+ < PET+/plasma+: Z = -2.863, *p* = 0.012

^d^ADNI MEM: PET-/plasma- < PET+/plasma+: Z = 4.341, *p* < 0.001

^e^ADNI EF: PET-/plasma- < PET+/plasma+: Z = 3.998, *p* < 0.001

^f^MMSE: PET-/plasma- < PET+/plasma+: Z = 4.716, *p* < 0.001

^g^CDR: PET-/plasma- < PET+/plasma+: Z = -3.743, *p* = 0.001; PET-/plasma+ < PET+/plasma+: Z = -2.302, *p* = 0.063

^h^CDR SB: PET-/plasma- < PET+/plasma+: Z = -3.869, *p* = 0.001; PET-/plasma+ < PET+/plasma+: Z = -2.769, *p* = 0.016

^i^ADNI MEM: PET-/plasma- < PET-/plasma+: Z = 2.415, *p* = 0.047, PET-/plasma- < PET+/plasma+: Z = 4.531, *p* < 0.001

^j^ADNI EF: PET-/plasma- < PET-/plasma+: Z = 3.878, *p* = 0.001; PET-/plasma- < PET-/plasma+: Z = 2.525, *p* = 0.023; PET-/plasma- < PET+/plasma-: Z = 2.840, *p* = 0.013

Abbreviation: ADNI EF, Alzheimer’s Disease Neuroimaging Initiative composite score of executive function; ADNI MEM, Alzheimer’s Disease Neuroimaging Initiative composite score of memory; CDR, clinical dementia rating; CDR SB, clinical dementia rating sum of boxes; IA-Elc, Elecsys immunoassay from Roche Diagnostics; IP-MS-WashU, immunoprecipitation followed by mass spectrometry method developed at Washington; IQR, interquartile range; MMSE, mini mental state examination; PET, positron emission tomography.
