# Supplementary material for: Characteristics of discordance between amyloid positron emission tomography and plasma amyloid-β 42/40 positivity
Source: Transl Psychiatry. 2024 Feb 10;14:88. doi: 10.1038/s41398-024-02766-6 (PMC10858862; doi:10.1038/s41398-024-02766-6)
Supplement: Supplementary file 4 — Supplementary table 4. PRS comparison of PET/plasma groups [file 41398_2024_2766_MOESM4_ESM.docx]

Supplementary table 4. PRS comparison of PET/plasma groups

|  | IP-MS-WashU | | | | | IA-Elc | | | | |
| --- | --- | --- | --- | --- | --- | --- | --- | --- | --- | --- |
|  | PET-/  plasma- | PET-/  plasma+ | PET+/  plasma- | PET+/  plasma+ | *p*-value | PET-/  plasma- | PET-/  plasma+ | PET+/  plasma- | PET+/  plasma+ | *p*-value |
| PRS^a^ | -0.3  (-0.66, 0.03) | 0.05  (-0.93, 0.68) | -0.55  (-0.59, 0.64) | 0.3  (-0.31, 1.31) | 0.214 | -0.53  (-0.85, 0.68) | -0.04  (-0.46, 0.55) | 1.82  (0.38, 1.88) | 0.12  (-0.54, 1.3) | 0.294 |

Data are shown as median (IQR).

^a^PRS were available only for 64 participants.

Abbreviation: IA-Elc, Elecsys immunoassay from Roche Diagnostics; IP-MS-WashU, immunoprecipitation followed by mass spectrometry method developed at Washington; IQR, interquartile range; PET, positron emission tomography; PRS, polygenic risk score.
