# Supplementary material for: Characteristics of discordance between amyloid positron emission tomography and plasma amyloid-β 42/40 positivity
Source: Transl Psychiatry. 2024 Feb 10;14:88. doi: 10.1038/s41398-024-02766-6 (PMC10858862; doi:10.1038/s41398-024-02766-6)
Supplement: Supplementary file 6 — Supplementary figure 2. PRS according to PET/plasma groups [file 41398_2024_2766_MOESM6_ESM.pdf]

Supplementary figure 2. PRS according to PET/plasma groups

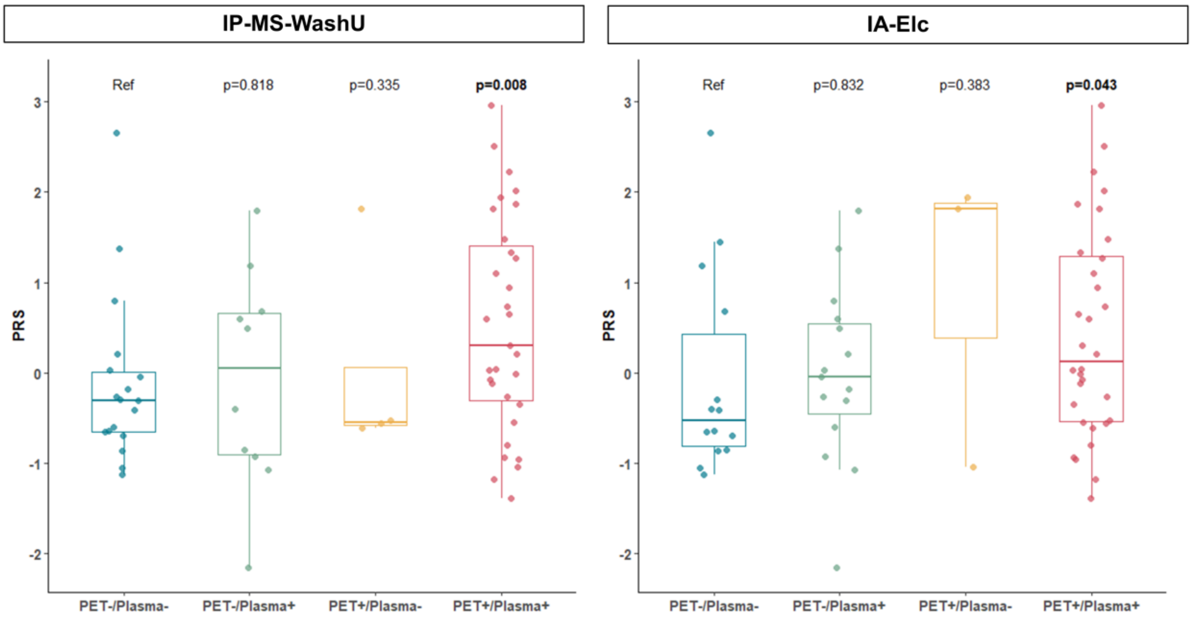

Association between PRS and PET/plasma groups were analyzed using multiple linear regression model adjusted for age and sex.

Abbreviations: IA-Elc, Elecsys immunoassay from Roche Diagnostics; IP-MS-WashU, immunoprecipitation followed by mass spectrometry method developed at Washington; PET, positron emission tomography; PRS, polygenic risk score.
